# Supplementary material for: Genome-Wide Analysis of the Soybean Calmodulin-Binding Protein 60 Family and Identification of GmCBP60A-1 Responses to Drought and Salt Stresses
Source: Int J Mol Sci. 2021 Dec 16;22(24):13501. doi: 10.3390/ijms222413501 (PMC8708795; doi:10.3390/ijms222413501)
Supplement: Supplementary file 1 [file ijms-22-13501-s001.zip › Supplemental.pdf]

Table S1. The values of expression patterns of GmCBP60 genes identified in the soybean genome under different treatments.

Gene ID	Gene Name	CK[1]-FPKM[2]	Drought[3]-FPKM	Salt[4]-FPKM
LOC100804548	CBP60A-1	5.825	42.395	26.365
LOC100793156	CBP60A-2	12.63	39.935	36.03
LOC100797598	CBP60B-1	0.35	28.045	14.63
LOC100812605	CBP60B-2	8.36	75.205	27.19
LOC100806393	CBP60B-3	2.555	12.22	4.515
LOC100790087	CBP60B-4	31.16	26.12	28.865
LOC100803029	CBP60B-5	27.12	59.04	35.89
LOC100798493	CBP60B-6	0.345	1.37	0.5
LOC100802994	CBP60C-1	8.915	28.275	14.315
LOC100816427	CBP60D-1	0.375	77.565	17.205
LOC100805759	CBP60D-2	0.41	83.32	10.075
LOC102664843	CBP60E-1	1.805	13.58	5.23
LOC100808358	CBP60E-2	1.345	8.475	5.025
LOC100775528	CBP60E-3	2.9	35.465	17.035
LOC100806598	CBP60G-1	0.075	6.435	2.775
LOC100792405	SARD1-1	0.075	2.71	0.765
LOC100788183	SARD1-2	1.59	108.685	14.93
LOC100803213	SARD1-3	0.265	32.95	6.43
LOC100802257	SARD1-like	0.675	254.035	12.89

[1] CK: control check.

[2] FPKM: fragments per kilobase of transcript per million mapped reads.

[3] Drought: drought treatment.

[4] Salt: salt treatment.

Table S2. The primers and sequences used for the experiments. The restriction sites were underline.

Primer Name	Primer Sequence
CBP60A-1-F	GTCGTGGTTTCCCTTGCCC
CBP60A-1-R	CATTGTCCGAGTTTGTTTGCG
CBP60A-2-F	TCTGTGACACTGATTCAATGGCT
CBP60A-2-R	CCTGTATTTGGTTTCCTCGTTT
CBP60B-1-F	TGTGCTTCGTGAATAAACTCCC
CBP60B-1-R	GCCCCTTCCCCTCTCTTGGT
CBP60B-2-F	TGTTGAAGCCCTGAAGGTTGAC
CBP60B-2-R	GCCTTCTATCCGCTTTGGG
CBP60B-3-F	GACCTGCGTTGGCTAGTGTA
CBP60B-3-R	GCCATCAGGGCCTTCTATCC
CBP60B-4-F	ATCAGCCCGAACGAAAGAGG
CBP60B-4-R	AAGAGACAGCCGGGACCTAA
CBP60B-5-F	GAGTTTCGTCTTTTCGGGCG
CBP60B-5-R	TTGCGTCGGTTGTTGGTTTC
CBP60B-6-F	GAGGGTGATGAAAGGGGTGTT
CBP60B-6-R	GATAGGGTTTGGTTCCCGATA
CBP60C-1-F	TACACGACAACCAGCCACTC
CBP60C-1-R	ATGAGATTGCCCTCCTCCCA
CBP60D-1-F	CTTGAGTTGTTCATTTGCTTTGTAA
CBP60D-1-R	GCCACTGATTGATGCTTGTTTT
CBP60D-2-F	AGGCAGTGGCAAAGTTCCAA
CBP60D-2-R	TTTGTTCCACCACCTCGCTT
CBP60E-1-F	GAATGCAAACTTCCAAGACCAA
CBP60E-1-R	CAGAACTCACCATCAAGGACACA
CBP60E-2-F	AGAAATGCTCAAGGTTTGGAAGA
CBP60E-2-R	CCTGGTCCTCGCTGAATGAA
CBP60E-3-F	GACATTCAACGAGGATCAGGGA
CBP60E-3-R	TCCAAAAGCAATATCAGCAAGC
CBP60G-1-F	GCTCTTACCACTTAGTTTGCGTTA
CBP60G-1-R	TGACCTCTACCATCAGCACCCT
GmAnnexin1-F	AGGTTGGGGAACTAACGAGG
GmAnnexin1-R	GTGTCCAAAGATGAACCAGCC
GmCAX3-F	TATTTCTGCACACCAAGGCT
GmCAX3-R	AAGACGTGGAGGACATGCTG
GmCBL4-F	TAGGTTGTATGATCTGAGGCAG
GmCBL4-R	TGACTCTTCAATATCCGTTCCTGT
GmCIPK24-F	GGTGTTTGAAGTTGCGCCAT
GmCIPK24-R	GCTTTCAGCCTTTCAGTGCT
GmDREB2-F	GAAAGCAGCAGCACCAACAA
GmDREB2-R	GGCGTAAGAACCCAACCAGA
GmERD1-F	CGTCCAGAATTGCTCAACAG
GmERD1-R	TGGGGTTATAGCCTTGTTGG

Table S2. The primers and sequences used for the experiments. The restriction sites were underline.

Primer Name	Primer Sequence
GmMYB118-F	TTGCTTGAGATTATGGTCCTTG
GmMYB118-R	CATCACCCTTTCCTTCAACC
GmMYB174-F	TGGCATAAAATAGGGAGCTGA
GmMYB174-R	AAAAGGCAAGTTCACGGATT
GmNAC11-F	TGCAAGGAGGAGCACAAGAG
GmNAC11-R	CACAGAACCCAGTCGTCCAA
GmNHX1-F	GGGTAGTGAGAACGCTGTG
GmNHX1-R	ATTGTGAGATGTTTGCTGGTAATC
GmRD20A-F	GTGGCACATGACTGAAGGAA
GmRD20A-R	ATCTTTCCAGCAGCACCTCT
GmSOS1-F	TTGTGCTGCATTTCTTCGAG
GmSOS1-R	CGTGCTTCTTCTCCTTCCAC
SARD1-1-F	TTGCACCAATCGGGGACATT
SARD1-1-R	GATCCTTGACAGCAAACGCC
SARD1-2-F	CAAGACAACAATAATAAGCCACCA
SARD1-2-R	GCACACTAAAATGCCTCATCACTC
SARD1-3-F	CTGGCGGAAAACGGATGA
SARD1-3-R	CAAAGTTGAAGGTTGTTGCTGAT
SARD1-like-F	TTCAGAGTTGCTGTTAGGGTGG
SARD1-like-R	CGTCATTGAGCTTAGGTGGGT
Soby Actin-RT-F	ACATTGTTCTTAGTGGTGGCT
Soby Actin-RT-R	CTGTTGGAAGGTGCTGAG
CBP60A1-1302-F	GGGACTCTTGACCATGATGTCGCTCAAGCGCCCC
CBP60A1-1302-R	TCAGATCTACCCATGGTCACAACCTTACCTGTATTTGA
CBP60A1-3301-F	GGACTCTTGACCATGATGTCGCTCAAGCGCCCC
CBP60A1-3301-R	ATTCGAGCTGGTCACCTCACAACCTTACCTGTATTTGA
CBP60A1-16318-F	TATCTCTAGAGGATCCATGTCGCTCAAGCGCCCC
CBP60A1-16318-R	TGCTCACCATGGATCCCAACCTTACCTGTATTTGA
CBP60A1-RNAi	CTCCGCATCGTCGACCTCATCGGAAACCGCCTCTCCGGC<br>TCCATTCCCGCCGGCATCGGCAGACTCCATCGGCTCACG<br>GTGCTCAACGTCGCCGACAATCTCATCTCCGGAACAATC<br>CCAACCTCGCTGGCGAACCTCAGCAGTCTCATGCACTTG<br>GATCTCCGAAACAACCTCTTCTCCGGGCCCATTCCAAGA<br>AACTTCGATCCGATCGAAAAACGGGAGTCTGCCCCTAA<br>GACAGATAAGCCGCCAAGAAGGCGCAAGTCAACCGCGA<br>GTTGTTGTATCATATCTACTGACAAAGATCACAAATGGG<br>ATGGCTGATTAGATACCTTGGCCTCCCAGATCGATTCGA<br>AGTTTCTTGGAATGGGCCCGGAGAAGAGGTTGTTTCGGA<br>GATCCAAGTGCATGAGACTGCTGAGGTTCGCCAGCGAGG<br>TTGGGATTGTTCCGGAGATGAGATTGTCGGCGACGTTGA<br>GCACCGTGAGCCGATGGAGTCTGCCGATGCCGGCGGGAA<br>TGGAGCCGGAGAGGCGGTTTCCGATGAGGTCGACGATGC

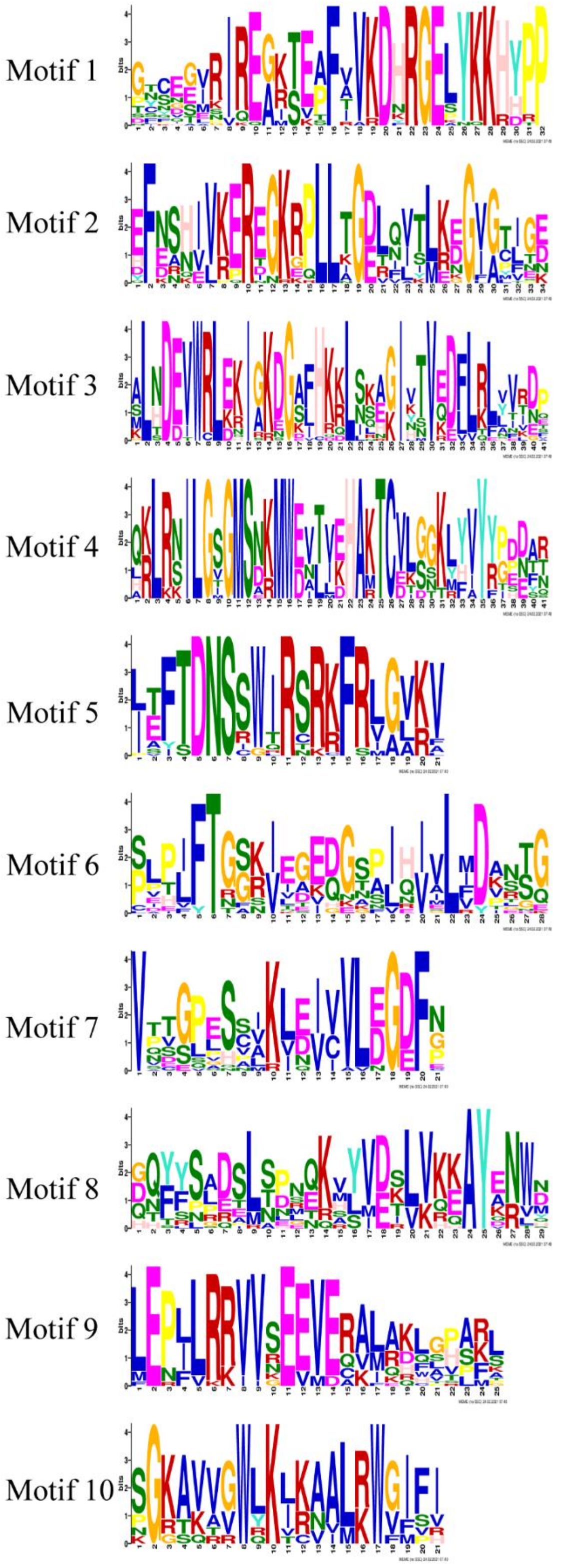

Figure S1. Sequence logo of global multiple alignment of GmCBP60s proteins by MEME program.

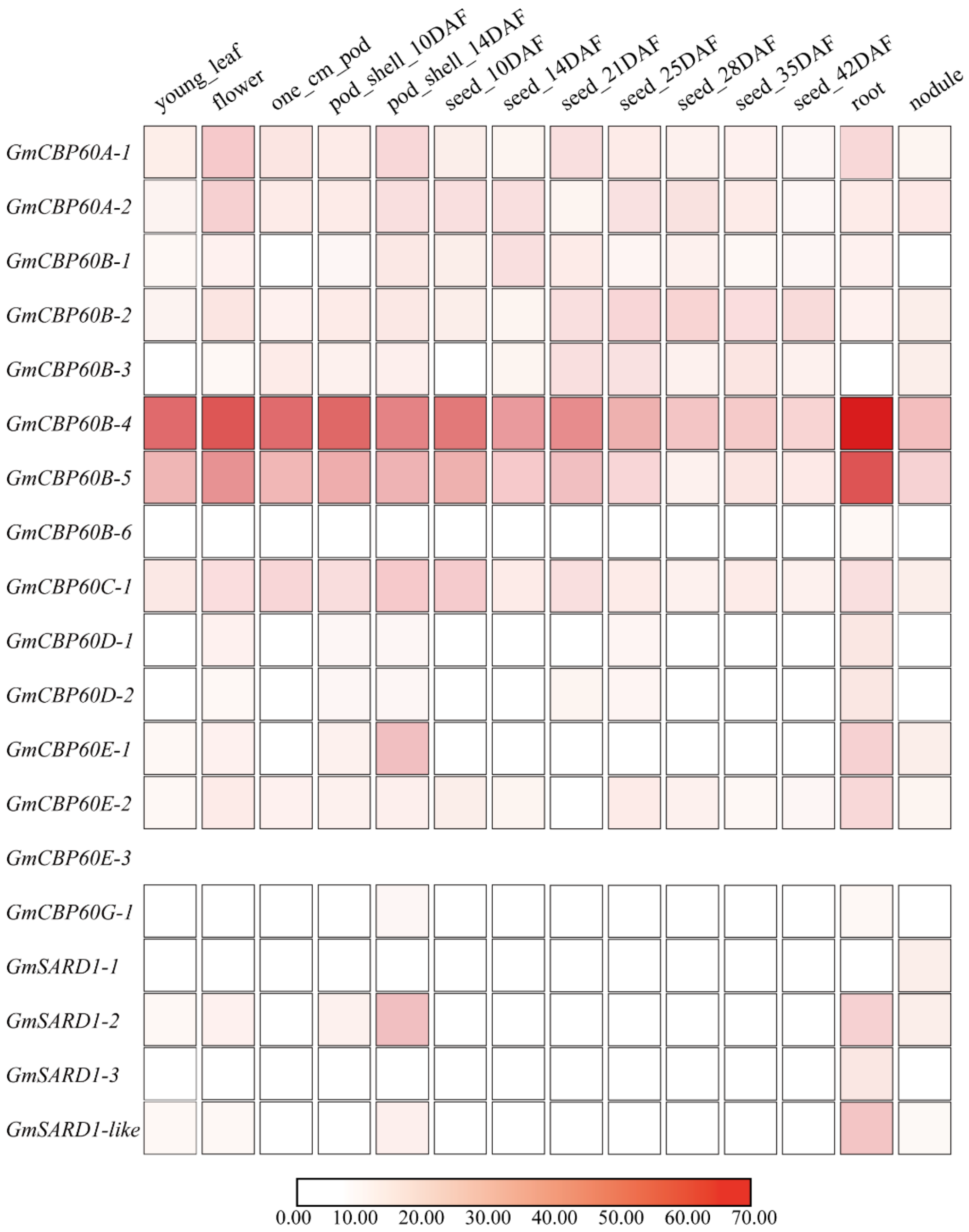

Figure S2. Quantified prediction of tissue expression in soybean. The deeper color signifies stronger expression.

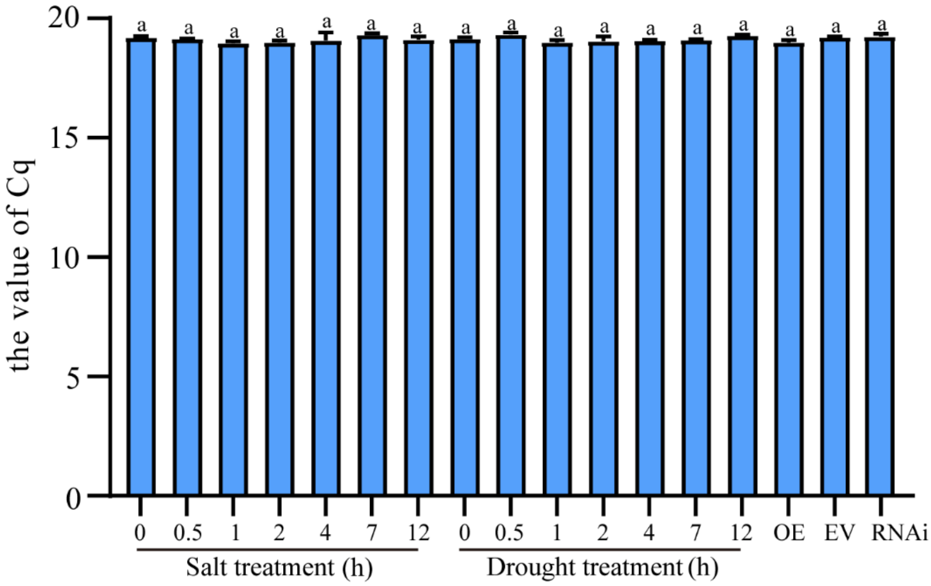

	1	2	3	4	5	6	7	8	9	AVERAGE
Salt-0 h	19.11131	19.11162	19.11157	19.25015	19.25940	19.25991	19.19152	19.20901	19.20310	19.18973
Salt-0.5 h	19.10836	19.11186	19.12801	19.15215	19.15226	19.15884	19.13627	19.13648	19.13759	19.13576
Salt-1 h	18.94419	19.09956	18.88718	19.00223	19.01590	19.01100	18.90250	18.91040	18.91568	18.96540
Salt-2 h	18.93865	18.90521	18.99986	19.10253	19.10359	19.10339	18.92110	18.92570	18.93010	18.99224
Salt-4 h	18.89975	18.89851	18.89746	19.03507	19.01576	19.09888	18.95420	18.92113	19.93962	19.07338
Salt-7 h	19.34762	19.24284	19.29196	19.38387	19.40721	19.38353	19.25692	19.25066	19.26197	19.31406
Salt-12 h	19.14933	19.16662	18.92677	18.92173	19.06099	19.08506	19.22160	19.20189	19.26786	19.11132
Drought-0 h	19.16285	19.16639	19.16877	19.20483	19.20116	19.20457	19.02684	19.05685	19.05489	19.13857
Drought-0.5 h	19.31050	19.35873	19.46529	19.35187	19.35926	19.35489	19.22156	19.21054	19.21891	19.31684
Drought-1 h	18.96093	18.92990	18.93927	19.11565	19.11893	19.11056	18.92568	18.93847	18.93025	18.99663
Drought-2 h	18.97697	18.97697	18.79666	19.15329	19.15698	19.49963	18.92634	18.92648	18.92889	19.03802
Drought-4 h	19.05083	19.04780	19.05648	19.12686	19.12657	19.12444	19.00363	18.99863	18.98563	19.05787
Drought-7 h	19.08234	19.08812	19.08342	19.13556	19.13005	19.13896	19.04649	19.05223	19.04848	19.08952
Drought-12 h	19.30934	19.33384	19.35337	19.22032	19.22649	19.22001	19.30616	19.28447	19.29465	19.28318
OE	18.97692	18.91269	18.93395	19.11363	19.13446	19.13767	18.90003	18.90663	18.91788	18.99265
EV	19.25135	19.18048	19.20420	19.22323	19.23116	19.26486	19.15626	19.15216	19.15249	19.20180
RNAi	19.33586	19.28745	19.25029	19.36229	19.33469	19.32774	19.10265	19.10488	19.10983	19.24619

$Max_{Cq}\text{-}Min_{Cq} < 0.5$

Figure S3. The analysis of Cq value about *β-actin* gene what is stably expressed in our material.

A

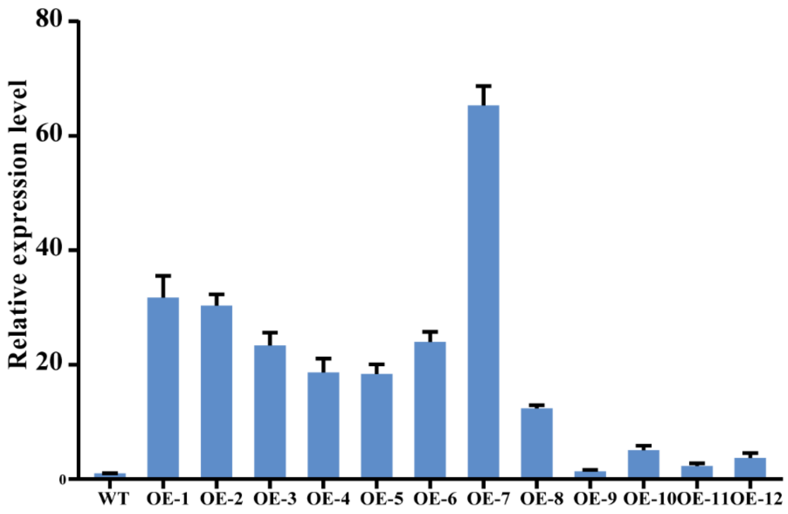

B

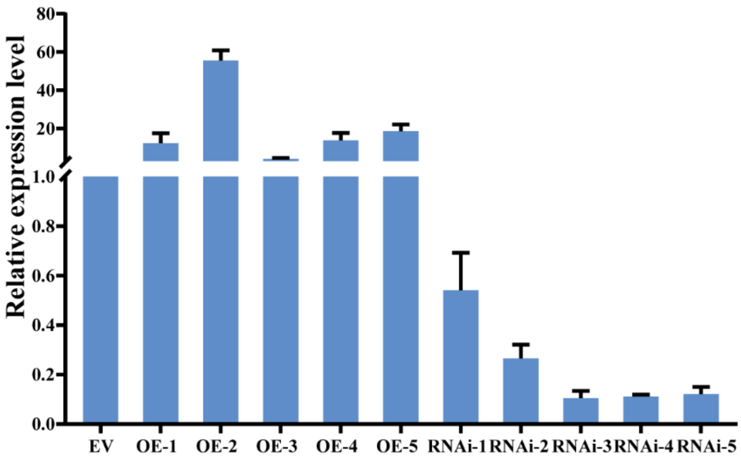

Figure S4. To confirmed relative expression level of *GmCBP60A-1* in transgenic *Arabidopsis* (A) and the hairy root soybean plants (B).
