# Supplementary figures and images for: Genome-Wide Analysis of the Soybean Calmodulin-Binding Protein 60 Family and Identification of GmCBP60A-1 Responses to Drought and Salt Stresses
Source: Int J Mol Sci. 2021 Dec 16;22(24):13501. doi: 10.3390/ijms222413501 (PMC8708795; doi:10.3390/ijms222413501)

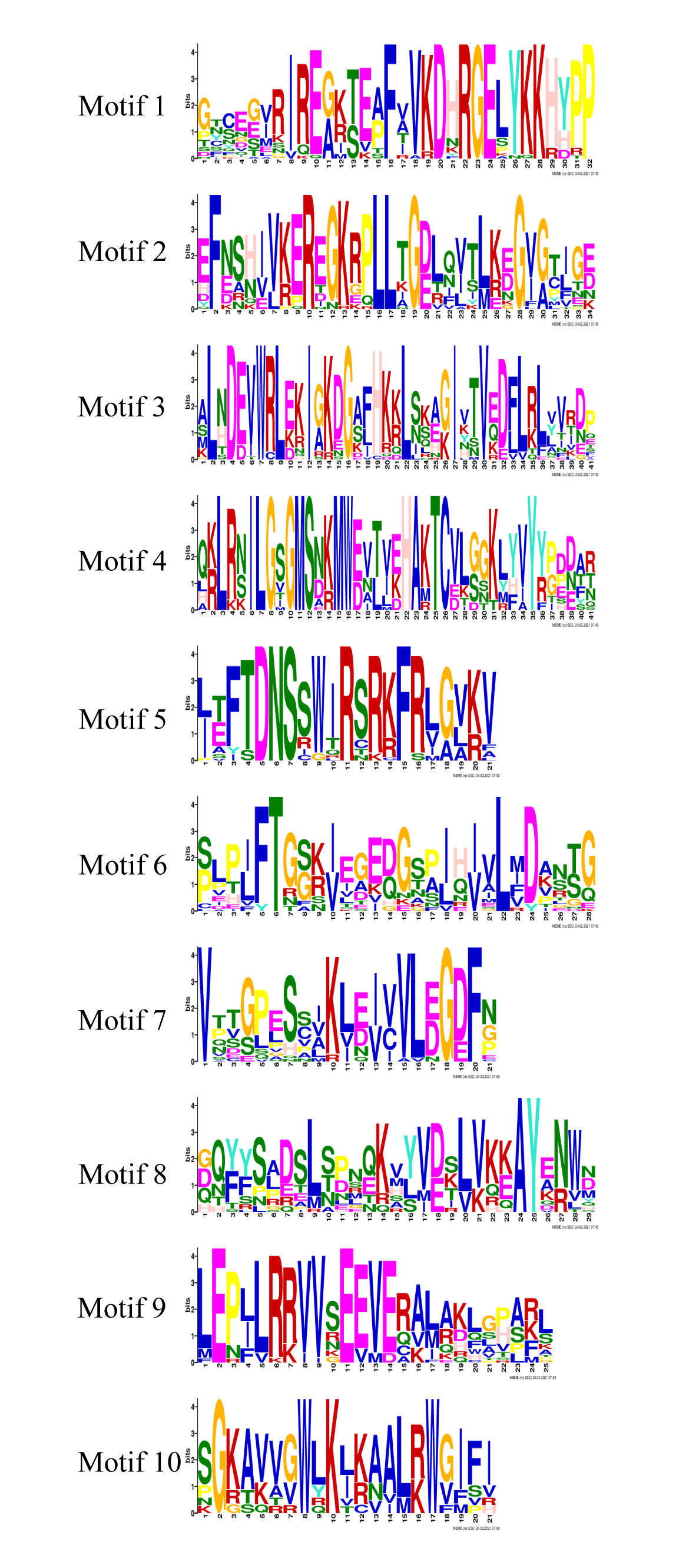

Supplement: Supplementary file 1 [file ijms-22-13501-s001.zip › Supplemental/Supplemental Figure S1.tif]

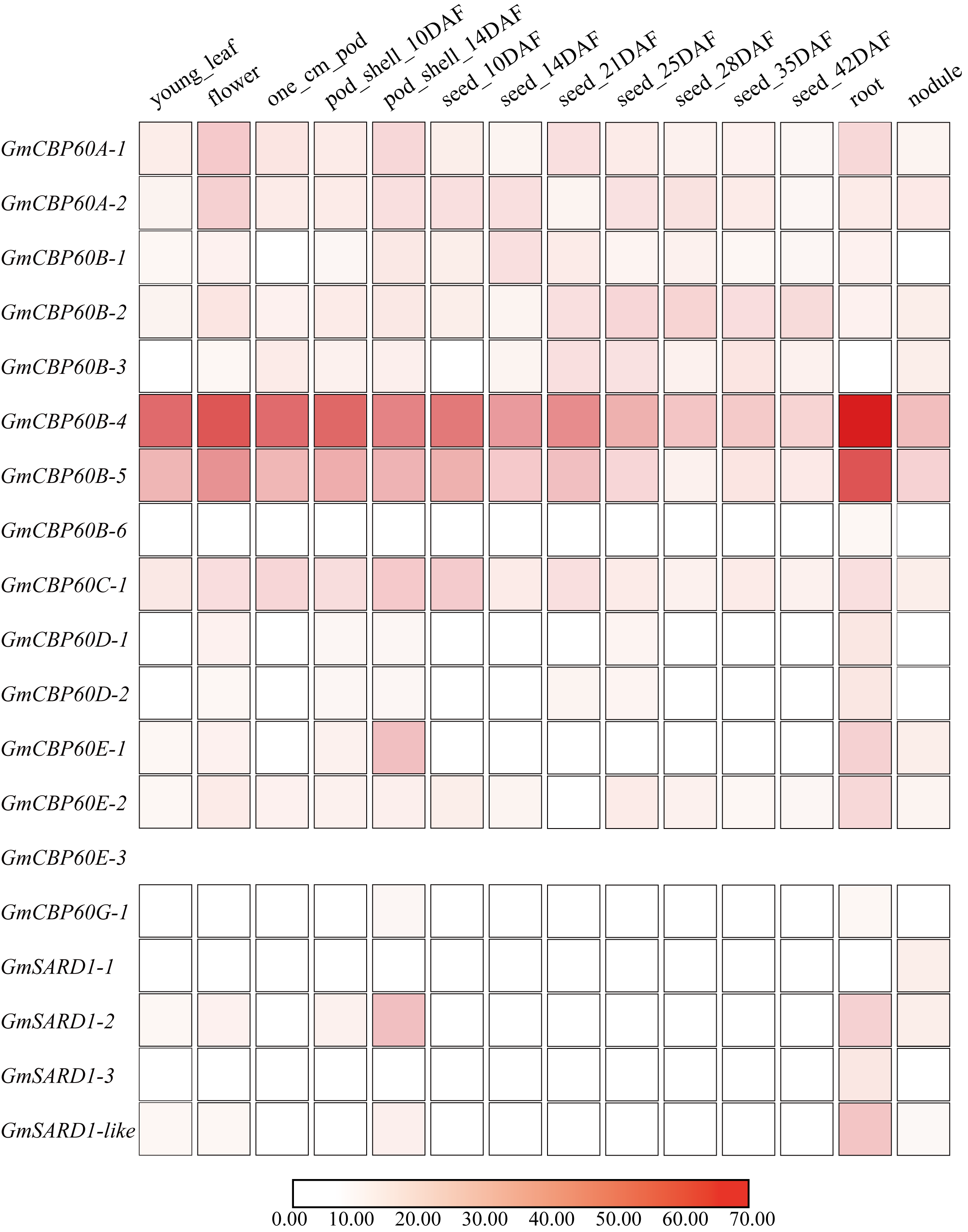

Supplement: Supplementary file 1 [file ijms-22-13501-s001.zip › Supplemental/Supplemental Figure S2.tif]

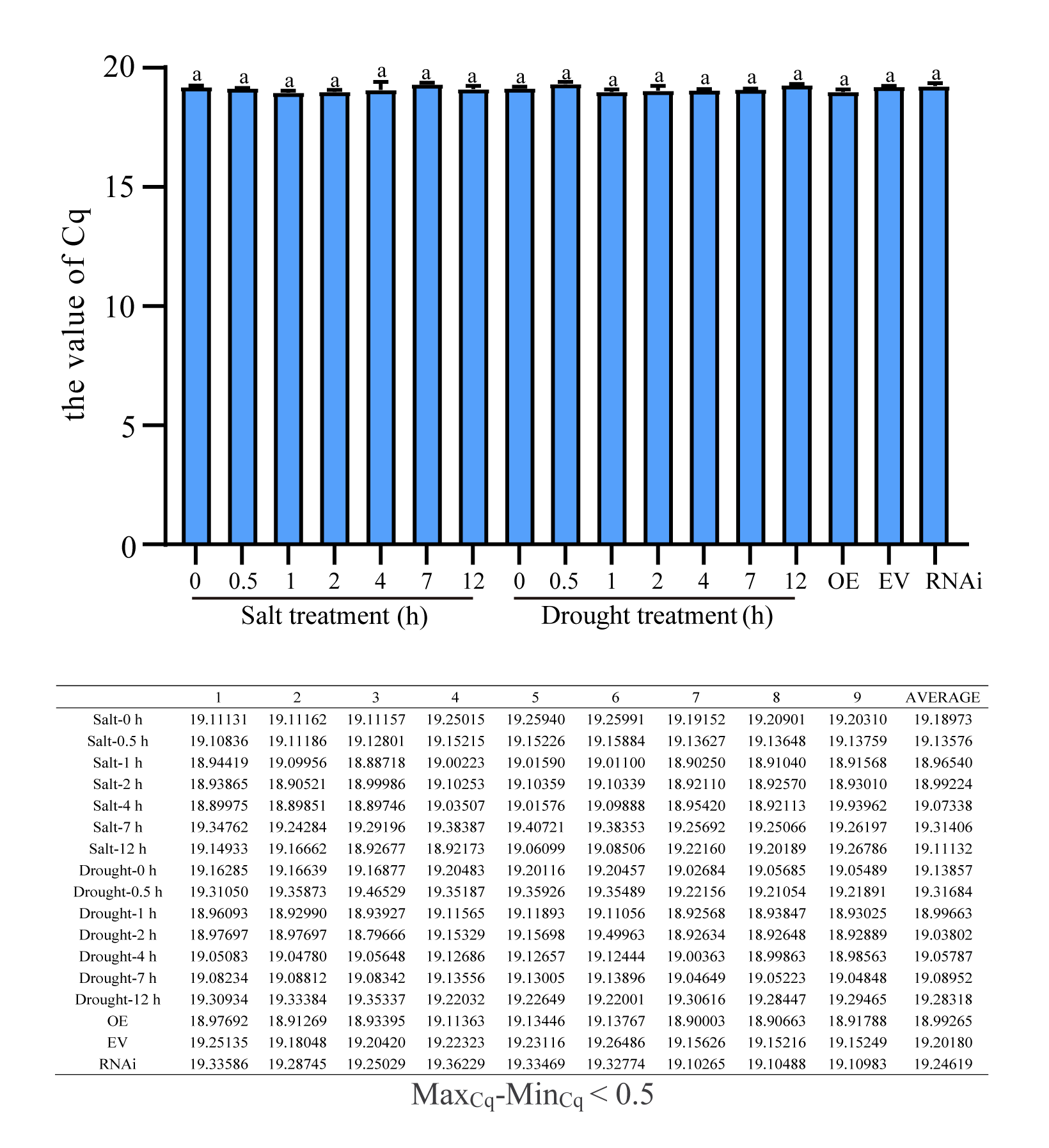

Supplement: Supplementary file 1 [file ijms-22-13501-s001.zip › Supplemental/Supplemental Figure S3.tif]

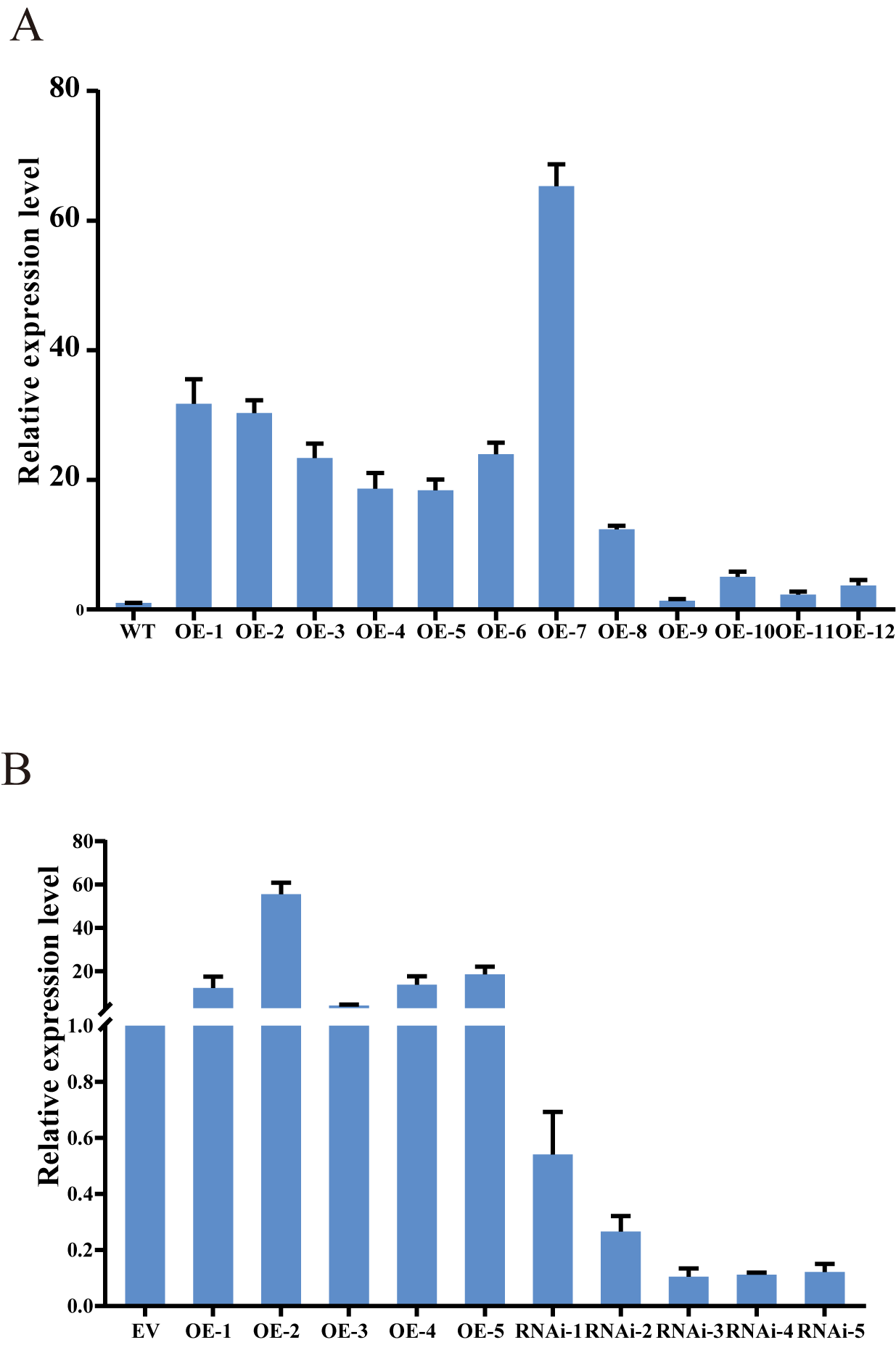

Supplement: Supplementary file 1 [file ijms-22-13501-s001.zip › Supplemental/Supplemental Figure S4.tif]
